# Olfactory receptor 78 modulates renin but not baseline blood pressure

**DOI:** 10.14814/phy2.15017

**Published:** 2021-09-21

**Authors:** Brian G. Poll, Jiaojiao Xu, Kunal Gupta, Tyler B. Shubitowski, Jennifer L. Pluznick

**Affiliations:** ^1^ Department of Physiology Johns Hopkins University School of Medicine Baltimore Maryland USA; ^2^ Oakland University William Beaumont School of Medicine Rochester Michigan USA

**Keywords:** blood pressure, olfactory receptors, renin

## Abstract

Olfactory receptor 78 (Olfr78) is a G protein‐coupled receptor (GPCR) that is expressed in the juxtaglomerular apparatus (JGA) of the kidney as well as the peripheral vasculature, and is activated by gut microbial metabolites. We previously reported that Olfr78 plays a role in renin secretion in isolated glomeruli, and that Olfr78 knockout (KO) mice have lower plasma renin activity. We also noted that in anesthetized mice, Olfr78KO appeared to be hypotensive. In this study, we used radiotelemetry to determine the role of Olfr78 in chronic blood pressure regulation. We found that the blood pressure of Olfr78KO mice is not significantly different than that of their WT counterparts at baseline, or on high‐ or low‐salt diets. However, Olfr78KO mice have depressed heart rates on high‐salt diets. We also report that Olfr78KO mice have lower renin protein levels associated with glomeruli. Finally, we developed a mouse where Olfr78 was selectively knocked out in the JGA, which phenocopied the lower renin association findings. In sum, these experiments suggest that Olfr78 modulates renin, but does not play an active role in blood pressure regulation at baseline, and is more likely activated by high levels of short chain fatty acids or hypotensive events. This study provides important context to our knowledge of Olfr78 in BP regulation.

## Graphical abstract

Olfactory receptor 78 (Olfr78) is a GPCR expressed in the kidney and peripheral vasculature which is activated by gut microbial metabolites. Using radiotelemetry, we determined that Olfr78KO mice do not significantly differ from their WT counterparts with regards to baseline blood pressure. However, we report that Olfr78KO mice have lower renin protein levels associated with glomeruli by immunofluorescence. These experiments suggest that Olfr78 status modulates renin, but does not play an active role in blood pressure regulation at baseline.

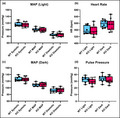

## 1 INTRODUCTION

Olfactory receptors (ORs) are the largest family of G‐protein coupled receptors (GPCRs), with nearly 350 genes in humans (Malnic et al., 2004) and nearly 1000 genes in mice (Godfrey et al., 2004). While they were initially characterized as specialized chemosensors in the nose, ORs are also expressed in a wide range of tissues where they play functional roles (Griffin et al., 2009; Spehr et al., 2003), including in the kidney (Pluznick et al., 2009). Although most murine ORs do not have a clear human ortholog, Olfactory receptor 78 (Olfr78) is one of only three murine genes to have a clear ortholog across 13 placental mammals, including humans (Niimura et al., 2014). In a prior study, we reported that Olfr78 localizes to both vascular smooth muscle cells and to the juxtaglomerular apparatus (JGA) of the kidney, where it plays a role in renin release (Pluznick et al., 2013). Both Olfr78 and its human ortholog OR51E2 respond to the short chain fatty acids (SCFAs) acetate and propionate (Pluznick et al., 2013), which are produced almost exclusively by commensal gut microbes (Perry et al., 2016). Studies in Olfr78KO mice indicated that Olfr78 is required for propionate‐induced renin release from ex vivo glomeruli, that Olfr78KO mice have lower plasma renin activity, and that they are hypotensive when blood pressure (BP) is measured under anesthesia via cannulation of the carotid artery (Pluznick et al., 2013).

A growing body of work by our group and others demonstrate that the gut microbiota influences host physiology, and that SCFA‐GPCR signaling is a key mechanism (Besten et al., 2013; Kimura et al., 2011; Onyszkiewicz et al., 2019; Pluznick et al., 2013). Olfr78 is expressed in both vascular smooth muscle and the JGA, which are both key contexts in the regulation of blood pressure. This fact, along with our prior work showing that Olfr78KO mice promote hypertensive responses to microbial metabolites(Pluznick et al., 2013), led us to hypothesize that Olfr78KO mice would have lower blood pressure at a conscious baseline and when blood pressure regulation was challenged by ingestion of differing salt diets. Thus, in this study we use radiotelemetry to measure BP and heart rate in Olfr78KO and Olfr78WT mice at baseline and under salt stress, and performed renin immunofluorescent (IF) staining to determine if the differences in plasma renin in Olfr78KO were mirrored by changes in renin at the JGA.

## 2 METHODS

### 2.1 Ethical approval

All animal protocols and procedures were approved by the Johns Hopkins Animal Care and Use Committee (accredited by the Association for the Assessment and Accreditation of Laboratory Animal Care).

### 2.2 Animals

Olfr78KO mice, initially generated by Bozza et al., (2009), were bred at Johns Hopkins and backcrossed for nine generations on C57Bl/6J. Olfr78 heterozygotes were crossed to obtain Olfr78KO and Olfr78WT mice. Mice were housed in individually ventilated cages with a maximum of five adult mice per cage. RenCre mice, which express cre recombinase instead of the *Ren1d* gene and are on a C57Bl6/J background, were a generous gift from Dr. Sequiera‐Lopez and Dr. Gomez (University of Virginia) and were bred at Johns Hopkins (Sequeira Lopez et al., 2004). Olfr78fl/fl mice (C57Bl6/J) were generated by our laboratory in collaboration with the Johns Hopkins Transgenic Mouse Core. Two loxP sites were inserted via CRISPR Cas9 flanking the *Olfr78* coding exon, which was confirmed by both PCR genotyping and Sanger sequencing (Figure S1, https://doi.org/10.6084/m9.figshare.15506130). Full excision of the Olfr78 gene was also confirmed by PCR genotyping and Sanger sequencing. For radiotelemetry studies, mice were housed separately in static cages placed on telemetry receiver platforms. Cages were autoclaved with corncob bedding, and animals were maintained on Teklad 2018SX, 18% protein diet. Animals were given water ad libitum using automatic watering systems or water bottles.

### 2.3 Radiotelemetry

Male and female Olfr78WT or Olfr78KO mice, 11–12 weeks of age, were implanted with radiotelemetry devices (PA‐C10, Data Science International), as described previously (Natarajan et al., 2016; Shubitowski et al., 2019), in order to measure blood pressure and heart rate. Radiotelemetry catheters were inserted into the right carotid artery, and the body of the transmitter was implanted subcutaneously in the abdomen. The procedure was performed under 2% isoflurane, and mice were given seven to fourteen days for recovery after surgery during which time mice were monitored for distress by recording weight, food/water intake, and behavior before any telemetry measurements were taken. BP was recorded continuously in the implanted mice after recovery. A 7‐day recording period was used to assess baseline BP. Systolic, diastolic, pulse pressure, and heart rate were averaged through this period.

### 2.4 Sodium loading studies

For sodium loading studies, mice were implanted with radiotransmitters to record blood pressure and maintained on a different control diet (0.49% NaCl, TD.110765), and BP was recorded for 7 days. This was followed by 14 days on a matched high‐salt diet (4% NaCl, TD.03095) and 14 days on a matched low‐salt diet (0.01% NaCl, TD.08290).

### 2.5 Renin immunofluorescence (IF)

10 micrometer thick cryosections were prepared from mouse kidneys that were fixed in 4% paraformaldehyde, followed by 30% sucrose. Sections were permeabilized with 1% SDS, blocked with 1% BSA 0.2% milk, followed by overnight incubation with a 1:200 dilution of rabbit anti‐renin antibody (a generous gift from Dr. Tadashi Inagami). Sections were then washed in TBS 0.1% BSA, and incubated with a 1:200 dilution of Alexa‐fluor 594 goat anti‐rabbit secondary followed by Hoechst staining. Sections were examined using a Keyence light microscope system. For renin‐glomeruli association, glomeruli were identified using Hoescht stain, then scored (+/‐) for proximity of renin IF signal. Three to five non‐sequential kidney sections per mouse were scored.

### 2.6 SCFA quantification

Blood from Olfr78KO and Ren1dCre78flfl was collected into K2‐EDTA tubes and spun down for plasma. Samples were then shipped for LC/MS analysis. The service was performed by the Mass spectrometry Core in the Research Resources Center of University of Illinois at Chicago.

### 2.7 Blood and urine chemistry

Whole blood samples were taken from the superficial temporal vein of 3‐month old male RenCreOlfr78^fl/fl^ and were analyzed using an iStat Chem8+ cartridge (Abbott). For urine electrolyte measurements, mice were placed into metabolic cages on a 0.49% “normal salt” diet (Envigo). After 3 days of acclimatization, urine was collected every 24 h and analyzed on an Easylyte Plus electrolyte analyzer (Medica).

### 2.8 Statistical analysis

Long‐term telemetry data were analyzed by two‐way ANOVA with Bonferroni's correction for multiple comparisons. Box‐and‐whisker plots show all points, with each point corresponding to one mouse averaged over the defined period, and span from minimum to maximum values. Significant differences between genotypes were defined by a Student's *t*‐test (in graphs with two sets of values) or one‐way ANOVA (for 3 or more sets of values) with a *p*‐value <0.05 vs. baseline. In‐text references to averages are presented as mean ± SEM.

## 3 RESULTS

### 3.1 Radiotelemetry implanted Olfr78KO mice do not exhibit significant MAP changes at baseline

**FIGURE 1:**
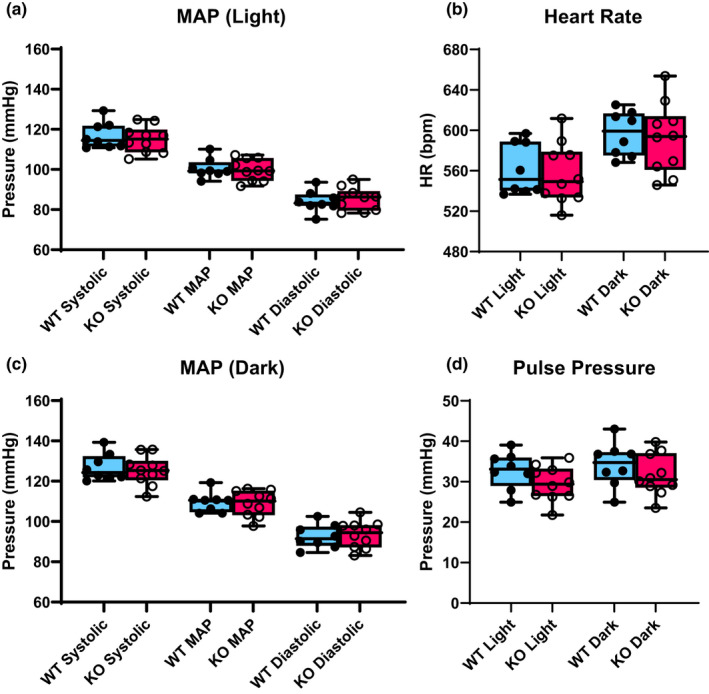
Olfr78KO mice exhibit normal blood pressure at baseline. Olfr78WT (closed circles) and Olfr78KO (open circles) mice were recorded and averaged for 7 days on a normal salt (0.49% NaCl) diet. Olfr78KO mice do not differ in terms of systolic, diastolic and mean arterial pressures during the light cycle (a) or the dark cycle (c). Heart rate (b) and pulse pressure (d) also did not differ. n = 8 WT, 10 KO

Prior studies of Olfr78KO mice, in which blood pressure was measured under anesthesia via carotid catheterization, implied that Olfr78KO are hypotensive (Pluznick et al., 2013). To test the hypothesis that Olfr78 are chronically hypotensive, we used radiotelemetry implanted mice to monitor blood pressure and heart rate in Olfr78WT and Olfr78KO mice. These mice were placed on a control “normal” salt diet (0.49% NaCl). Olfr78KO mice did not show any significant differences in BP at baseline compared to their WT counterparts. While on the normal diet for a 7 day baseline, Olfr78WT mice had an average light cycle mean arterial pressure (MAP) of 100.5 ± 1.7 mmHg and Olfr78KO mice had an average of 99.8 ± 1.7 mmHg (Figure 1a). Average dark cycle MAP was 109.6 ± 1.7 for Olfr78WT and 109.0 ± 2.0 for Olfr78KO mice (Figure 1c). The reported baseline values represent averages from a 7‐day recording in n = 8 Olfr78WT and n = 10 Olfr78KO mice. There were also no significant differences between genotypes for systolic pressure, diastolic pressure, heart rate (HR, Figure 1b), and pulse pressure (Figure 1d) after 7 days of a normal salt diet. These values are a combination of male and female data; although we are not well powered to measure females alone (n = 2 for Olfr78WT and n = 3 for Olfr78KO), there were no apparent differences between males and females.

### 3.2 Olfr78KO mice exhibit bradycardia under high‐salt stress

**FIGURE 2:**
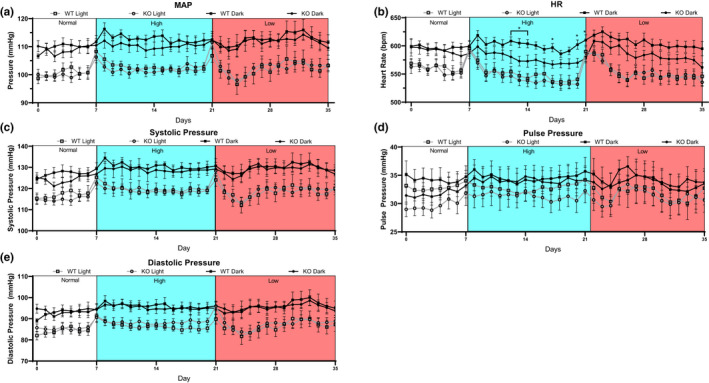
Chronic Blood pressure measurements in Olfr78KO mice. Olfr78WT (squares) and Olfr78KO (circles) mice were recorded for 7 days on a normal salt diet, 14 days on a high‐salt diet (4% NaCl, blue area) and 14 days on a low‐salt diet (0.01% NaCl, red area). Each day was split into a light cycle (open square/circle) and a dark cycle (closed square/circle) and averaged for mean arterial pressure (a), systolic pressure (b), diastolic pressure (c), heart rate (d), and pulse pressure (e). A significant depression in heart rate was seen during high‐salt diet on the dark cycle on days 12–14, 17, 20, and 21 (*p* < 0.05) n = 8 WT, 10KO. * compared to Olfr78WT by two‐way ANOVA

To further interrogate BP control in Olfr78KO mice, BP was measured after challenge with a high‐salt diet and a low‐salt diet. After a 7‐day baseline on a normal salt diet (0.49% NaCl, summary data shown in Figure 1), Olfr78WT and Olfr78KO mice were placed on a high‐salt diet (4% NaCl, 14 days), followed by a low‐salt diet (0.01% NaCl, 14 days). There were no significant differences in MAP, systolic, pulse, or diastolic pressure between Olfr78WT and Olfr78KO mice on high‐ or low‐salt diets (Figure 2a,c,d,e), although Olfr78KO did trend toward a lower MAP during the dark cycle on a high‐salt diet. While HR showed no differences based on genotype during the light cycle, HR was significantly depressed during the dark cycle in Olfr78KO mice (*p* < 0.05 on days 12–14,17, 20, and 21, *p* = 0.04 for 14‐day average HR of 601.4 ± 6.4 bpm vs. 577.2 ± 8.3 bpm, Olfr78WT vs. Olfr78KO, respectively, Figure 2b). This dark cycle HR depression was similar to a trend seen on low‐salt diet, though it was not significant (605.6 vs. 584.9 bpm Olfr78WT vs. Olfr78KO, respectively).

### 3.3 High‐ and low‐salt diets do not affect weight in Olfr78KO mice, but do affect plasma SCFA levels

**FIGURE 3:**
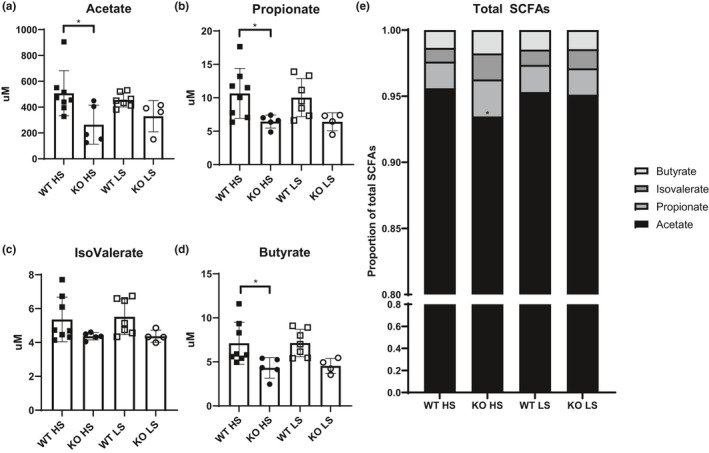
Effects of Salt Diet on plasma SCFAs in Olfr78KO mice. Cohorts of Olfr78WT (squares) and Olfr78KO mice (circles) were placed on high‐ and low‐salt diets for 7 days. At the end of the 7 days, plasma was harvested and sent for LC/MS analysis of SCFA composition (a‐e). Acetate was significantly decreased in Olfr78KO mice on high‐salt diet (HS, *p* = 0.02 vs. Olfr78WT), but not significantly so on a low‐salt diet (LS) (a). Propionate was also significantly decreased in Olfr78KO on high‐salt diet (*p* = 0.03 vs. Olfr78WT) (b). There were no differences in the levels of Isovalerate (IsoVal) (c). Butyrate (But) was also significantly decreased in Olfr78KO on high‐salt diet (*p* = 0.02 vs. Olfr78WT) (d). All SCFAs shown as a proportion of total SCFAs is shown in (e). * *p* < 0.05 by one‐way ANOVA

To provide additional context to our high‐ and low‐salt telemetry data, a separate cohort of Olfr78WT and Olfr78KO mice were placed onto either a high‐ or low‐salt diet, and at the end of one week plasma was collected for SCFA measurements. The SCFAs acetate and propionate are ligands for Olfr78, and changes in these SCFA levels could be an additional variable in any Olfr78‐dependent effects we observe (Pluznick et al., 2013). Olfr78KO mice on a high‐salt diet showed significantly lower levels of plasma acetate when compared to Olfr78WT mice on a high‐salt diet (507.4 ± 61.8 µM vs. 263.7 ± 67.6 µM Olfr78WT vs. Olfr78KO, respectively, Figure 3a). This is also a significant decrease in acetate as a proportion of total plasma SCFAs (Figure 3e). Olfr78KO mice also had significantly lower levels of plasma propionate compared to Olfr78WT mice on the high‐salt diets (10.6 ± 1.3 µM vs. 6.4 ± 1.0 µM (Figure 3b)); however, this was not significantly different when measured as a proportion of total SCFAs. There were no genotypic differences with regards to plasma isovalerate levels (Figure 3c), but plasma butyrate was lower in Olfr78KO compared to Olfr78WT mice on the high‐salt diet (7.1 ± 2.3 µM vs. 4.3 ± 1.1 µM (Figure 3d); neither isolvalerate nor butyrate are ligands for Olfr78. There were also no genotypic differences on either high‐ or low‐salt diets with respect to changes in weight (Figure S2, https://doi.org/10.6084/m9.figshare.15506130).

### 3.4 Olfr78KO mice display lower JGA‐localized renin levels

**FIGURE 4:**
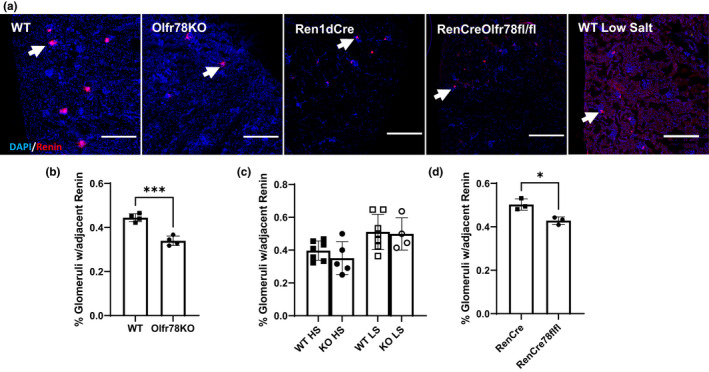
Renin association with the JGA in Olfr78KO mice. (a) Representative images of mouse kidneys showing renin staining (red) and nuclear clustering (blue) that indicates glomeruli (arrows) in WT, Olfr78 global KO, Ren1dCre, Ren1dCreOlfr78^fl/fl^, and WT on a low‐salt diet. Scale bars = 500µm. (b) Olfr78KO mice (circles) have less renin signal association with glomeruli when compared to Olfr78WT(squares) (*p* = 0.0003, n = 4 mice for each genotype WT and Olfr78 whole body KO, Total individual glomeruli counted n = 1921 WT n = 1897 Olfr78KO). (c) Though this trend of lower renin association was present on high‐ and low‐salt diets (HS and LS, respectively), there were no significant differences between genotypes (n = 7 WT HS, n = 5 KO HS, n = 7 WT LS, n = 4 KO LS, total individual glomeruli counted n = 135 WT HS, n = 106 KO HS, n = 151 WT LS, n = 75 KO LS) (d). Mice where Olfr78 is selectively knocked out in the JGA (ReninCreOlfr78^fl/fl^) also have significantly lower renin association when compared to control mice. (*p* = 0.02, n = 3 mice for each genotype RenCre78+ and RenCre78flfl, Total individual glomeruli counted n = 1002 RenCre78+ n = 817 RenCre78flfl. Three to five non‐sequential kidney sections analyzed for each mouse. ***p < 0.001, *p < 0.05 by student's *t*‐test

We previously reported that Olfr78KO mice have lower plasma renin activity and hypothesized that this resulted from the deletion of Olfr78 from the JGA. To better understand this potential interaction, we stained kidney cryosections from Olfr78KO and Olfr78WT mice and stained them for renin via immunofluorescence (IF). We reasoned that if renin expression extends along a longer length of the afferent arteriole, then a larger % of glomeruli in a given cryosection would have adjacent renin stain. Thus, we used the % of glomeruli with adjacent renin stain as an index of changes in renin protein expression. Using the Hoechst nuclear stain, we identified glomeruli in the kidney sections due to the characteristic clustering of cell nuclei. We then determined whether or not there was renin IF signal associated with each glomerulus (Figure 4a). Olfr78KO kidney sections show a significant reduction in the percentage of glomeruli that are associated with a renin IF signal (Figure 4b).

This analysis was repeated for mice that had undergone high‐ and low‐salt diets. While the trend of lower JGA associated renin signal is still present for the HS diet, there are no significant genotypic differences between Olfr78WT and Olfr78KO on high‐ and low‐salt diets (Figure 4c).

### 3.5 Confirming renin association phenotype in mice where Olfr78 is selectively knocked out in the JGA

Initial studies of Olfr78KO mice found that Olfr78 is expressed in at least two locations which influence blood pressure regulation: the JGA, and vascular smooth muscle cells in the periphery (Pluznick et al., 2013). To determine if the change in renin staining observed in the whole‐animal KO (Figure 4a,b) is due to Olfr78 expression in the JGA, we crossed Olfr78^fl/fl^ to a mouse where Cre recombinase is expressed under the renin *Ren1d* promoter (RenCre) to eliminate Olfr78 expression in renin‐expressing cells, including the JGA (RenCreOlfr78^fl/fl^). We then repeated the same analysis, staining kidney cryosections from Ren1dCre and RenCreOlfr78^fl/fl^ mice for renin via immunofluorescence (IF). These mice were on a regular salt diet. RenCreOlfr78^fl/fl^ kidney sections had a reduction in the percentage of glomeruli associated with renin IF signal compared to RenCre controls (Figure 4d).

To confirm normal physiology and renal electrolyte clearance, whole blood samples were taken from RenCre and RenCreOlfr78^fl/fl^ and 24‐h urine samples were taken from RenCre and RenCreOlfr78^fl/fl^ mice placed in metabolic cages. No significant differences were found between genotypes for blood chemistry (Table S1, https://doi.org/10.6084/m9.figshare.15506130) or for 24‐h urine electrolyte excretion (Figure S3, https://doi.org/10.6084/m9.figshare.15506130).

## 4 DISCUSSION

In this study, we demonstrate that Olfr78KO mice have normal BP at baseline and that Olfr78KO mice are not salt‐sensitive. However, we find that Olfr78KO correlates with defects in renin association with the JGA; this finding was replicated in a JGA specific Olfr78KO mouse model. These data are in agreement with our previous report (Pluznick et al., 2013) of lowered plasma renin activity and decreased ex vivo renin release in whole‐animal Olfr78KO. Our studies provide valuable context to the function of Olfr78 in chronic BP regulation at baseline and under salt stress.

### 4.1 Olfr78 and blood pressure at baseline

Our studies demonstrate that Olfr78KO mice do not have significantly different BP at baseline when compared to their Olfr78WT counterparts as measured by the ‘gold standard’ of radiotelemetry. Our prior study on Olfr78KO mice suggested that these mice might be hypotensive (Pluznick et al., 2013). However, in the prior study mice were anesthetized, MAP was measured only at a single discrete point, and HR was not recorded. Anesthesia is known to cause myocardial depression (Constantinides & Murphy, 2016; Doursout & Chelly, 1988), as well as changes in vascular tone (Constantinides et al., 2011), hormonal release, and metabolism. Furthermore, the Olfr78KO mice, which were initially generated in a 129/B6 mixed background (Bozza et al., 2009), were not fully backcrossed into a C57BL/6 background at the time of the previous study. In addition, with any phenotype that is driven in part by gut microbial signals, there are a host of variables that can complicate its presentation. Microbial composition of mouse models can differ between institutions (Ivanov et al., 2008; Montonye et al., 2018; Rausch et al., 2016), and prior blood pressure studies with Olfr78KO were done at Yale University. Cage effects, bedding, and minor changes in dietary composition can also affect gut microbiota composition (Ericsson et al., 2018). Though we cannot directly trace any of our observations to changes in these variables, they are a notable caveat when comparing these data to prior studies.

While our results do not show a statistically significant difference in MAP at baseline, there is a downward trend in Olfr78KO MAP and systolic pressure during the dark cycle on a normal salt diet, with some separation at days 2–3 (difference of ~5–6 mmHg), as well as a downward trend in MAP during the dark cycle on a high‐salt diet. These data suggest that KO of Olfr78 only has a minor effect on MAP at a conscious baseline.

These findings are important given prior work from our laboratory showing isolated systolic hypertension in mice lacking Gpr41, another GPCR that responds to SCFAs and is expressed in the vasculature (Natarajan et al., 2016). This, along with the finding that Olfr78KO mice have an exaggerated hypotensive response to SCFAs, while Gpr41KO mice have a hypertensive response, led us to hypothesize that Olfr78 and Gpr41 have an opposing relationship, with Olfr78 working to raise blood pressure and Gpr41 working to lower blood pressure. Key to this hypothesis is the fact that Gpr41 has a much lower EC_50_ for propionate (EC_50_ = ~11 µM) (Brown et al., 2003) when compared to Olfr78 (EC_50_ ~0.9 mM for propionate) (Pluznick et al., 2013). Thus, we previously hypothesized that Gpr41 plays a role in setting baseline blood pressure, but that Olfr78 may only be activated when SCFA levels are high (Pluznick, 2016). Additionally, other GPCRs that respond to SCFAs include Gpr43 (Brown et al., 2003), Gpr109a (Taggart et al., 2005) and Olfr558 (Halperin Kuhns et al., 2019), all of which may also contribute to SCFA‐driven physiological responses. Our telemetry data further confirm that Olfr78 does not play a pivotal role in the maintenance of blood pressure under steady‐state conditions and may only become active at high plasma SCFA levels. Altering plasma SCFAs through dietary manipulations, long‐term infusions, or in the context of a leaky gut may reveal a more pronounced effect of the loss of Olfr78. It is also worth noting that SCFA levels differ in the Olfr78WT and Olfr78KO mice on the high‐salt diet, implying that the absence of a SCFA receptor alters either microbial production of SCFAs or host handling of SCFAs (we previously reported that the gut microbes themselves are similar in Olfr78WT and Olfr78KO) (Pluznick et al., 2013). While the significant changes in acetate and propionate could result in a difference in Olfr78 activation, the Olfr78 dependent difference in butyrate levels is intriguing because Olfr78 is not known to respond to butyrate (Pluznick et al., 2013). The impact of other butyrate sensitive GPCRs may be playing a role here, though further studies are needed to address this finding.

### 4.2 Salt sensitivity in Olfr78KO

We initially hypothesized that the low‐salt diet would exaggerate any hypotensive phenotype in the Olfr78KO. However, Olfr78KO mice do not show any significant changes compared to Olfr78WT mice in MAP, systolic, or diastolic pressure during high‐ and low‐salt diets. Interestingly, Olfr78KO mice have a significantly lower heart rate on a high‐salt diet. Long‐term telemetry recordings have shown decreases in HR upon high‐sodium diets (Carlson & Wyss, 2000), though this is attributed as a response to a corresponding increase in MAP. Other studies have shown no HR effect of sodium load (Bagnall et al., 2006; Escano et al., 2009; Kim et al., 2008). A drop in HR with no corresponding change in MAP could indicate a compensatory effect, which could indicate a degree of salt sensitivity. Additionally, LC/MS studies on plasma indicate that Oflr78KO mice have lower plasma acetate values under high‐salt diet, which could be another driving factor. The precise role of Olfr78 in this observed bradycardia or in lower plasma acetate levels on high‐salt diet will require further study.

### 4.3 Potential roles in other tissues

These studies were primarily undertaken using whole body Olfr78KO mice. Olfr78 is known to be expressed in the renal JGA, as well as the smooth muscle cells of the peripheral vasculature (Pluznick et al., 2013). Additionally, Olfr78 expression has been reported in colonic enteroendocrine cells, autonomic ganglia, and the carotid body (Chang et al., 2015; Fleischer et al., 2015; Weber et al., 2002), although Olfr78’s role in the carotid body has been a disputed topic (Chang et al., 2015; Peng et al., 2020; Torres‐Torrelo et al., 2018). Expression of Olfr78 in this context may be playing a role in the bradycardia we observe under salt stress. Although we cannot rule out the possibility that the absence of Olfr78 in multiple tissues in the whole‐animal KO may be obfuscating a potential tissue‐specific phenotype, future tissue‐specific knockout studies can help to address this point. The Olfr78flox mouse model is a potential tool to dissect tissue‐specific roles of Olfr78. To the best of our knowledge, the only context where renin and Olfr78 have overlapping expression is in the renal JGA, thus, we expect that Olfr78 is absent only in the JGA in the Ren1DCre‐Olfr78 flox mice.

### 4.4 Renin in Olfr78KO

We previously published that Olfr78KO mice have lower plasma renin activity at baseline, and that these mice are deficient in their ability to release renin from the JGA *ex vivo* (Pluznick et al., 2013). In agreement with these studies, we observe a significant decrease in renin IF signal associated with glomeruli in both whole‐animal Olfr78KO and in JGA specific renin knockouts. This further corroborates the hypothesis that Olfr78 may play a role in modulating renin or renin‐expressing cells. Studies with complete ablation of renin‐expressing cells show no difference in blood pressure among male mice, though female mice were hypotensive (Sequeira Lopez et al., 2004). This combined with our Olfr78KO data makes it highly unlikely that RenCreOlfr78^fl/fl^ mice would exhibit a blood pressure phenotype in telemetry studies. Though the change in renin IF signal is a promising observation, from these data alone we cannot determine if Olfr78 is playing a direct role on renin expression and release, or if there may be changes in JG cell development, differentiation, or indirect effects from other tissues. Future studies measuring renin levels in JGA specific knockout mice are required to definitively conclude if renin is directly modulated by Olfr78.

We report here that Olfr78KO mice show normal MAP at baseline and under salt stress. This is the first time that long‐term chronic blood pressure regulation has been studied in the context of Olfr78. This study suggests that Olfr78 plays a minimal role in the maintenance of chronic blood pressure, and may only become active under physiological stress. As the gut microbiota and their receptors become increasingly studied as a potential therapeutic target, a complete understanding of the roles of SCFA receptors is necessary. This study provides important context to our knowledge of Olfr78 and how dietary changes can impact host physiology.

## AUTHOR CONTRIBUTIONS

B.G.P., J.X., K.G., T.S., and J.L.P. participated in research design. B.G.P., J.X., K.G., and T.S. conducted experiments. B.G.P., J.X., K.G., T.S., and J.L.P. performed data analysis. B.G.P., J.X., T.S., and J.L.P. wrote or contributed to the writing of the manuscript.

## Supporting information



Supplementary Material
