# Supplementary material for: Olfactory receptor 78 modulates renin but not baseline blood pressure
Source: Physiol Rep. 2021 Sep 21;9(18):e15017. doi: 10.14814/phy2.15017 (PMC8455973; doi:10.14814/phy2.15017)
Supplement: Supplementary file 1 — Supplementary Material [file PHY2-9-e15017-s001.pdf]

Supplemental Data : **Olfactory Receptor 78 modulates renin but not baseline blood pressure**

Brian G. Poll, Jiaojiao Xu, Kunal Gupta, Tyler B. Shubitowski, Jennifer L. Pluznick

American Journal of Physiology – Renal Physiology

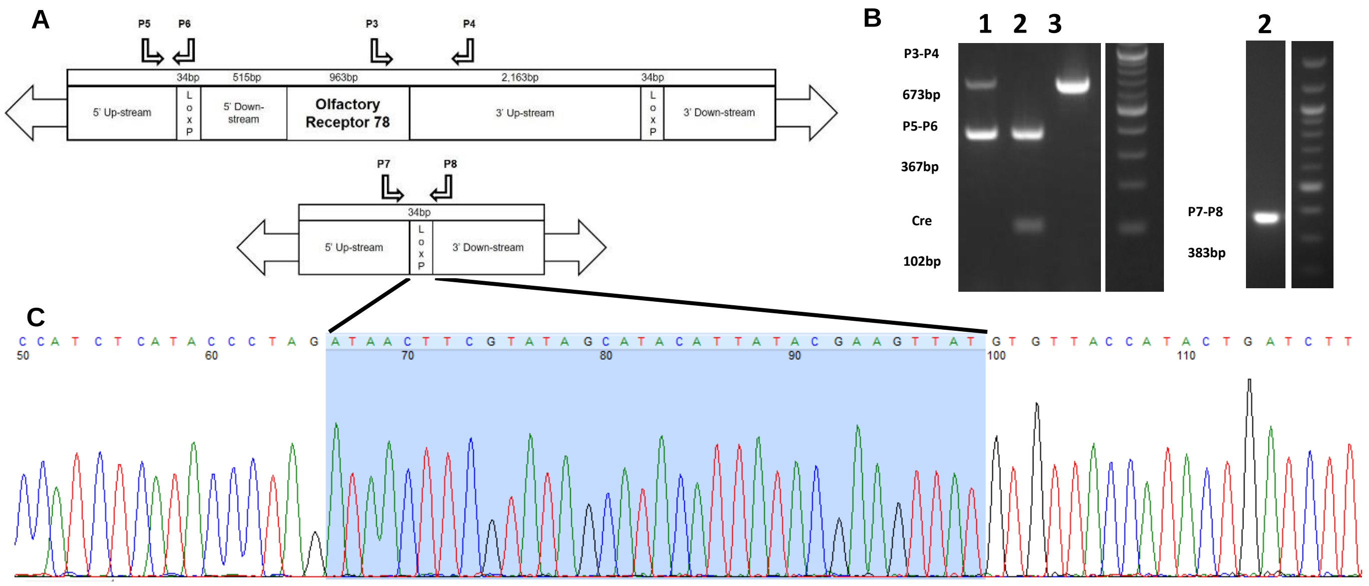

Supplemental Figure 1: Generation of the Olfr78$^{fl/fl}$ mouse. Using CRISPR, LoxP sites were inserted upstream and downstream of the *Olfr78* exon as shown (A). Primers P3-P4 were designed to detect the presence of the *Olfr78* gene, primers p5-p6 detect the presence of the LoxP site, and primers p7-p8 confirm *Olfr78* excision. Mice were screened by PCR (B) for LoxP insertion and *Olfr78* excision, which either showed Olfr78 and loxP (lane 1) or loxP and cre expression with Olfr78 excision (lane 2). Lane 3 shows a mouse with Olfr78 intact but no loxP insertion. *Olfr78* excision is shown with primers P7-P8, which yields a 383 bp band. *Olfr78* excision was confirmed by sanger sequencing (C).

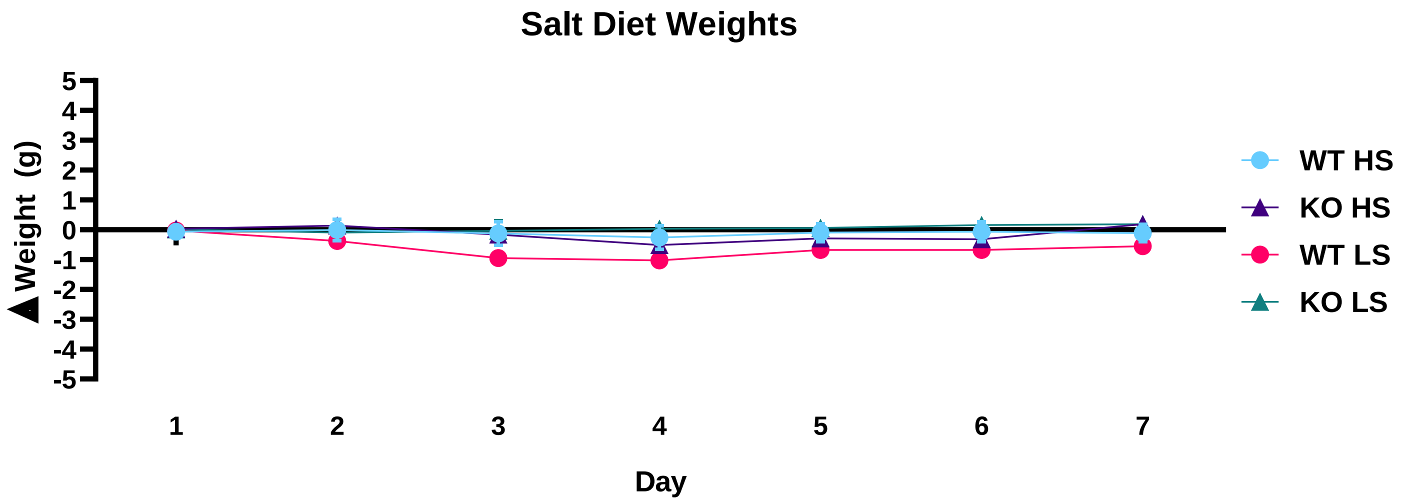

Supplemental Figure 2: Effects of Salt Diet on weight in Olfr78KO mice. Cohorts of Olfr78 WT (circle) and Olfr78 KO (triangle) mice were placed on high salt (HS) and low salt (LS) diets for 7 days and weight was recorded daily **(A)**. Daily weights are shown as Δ from baseline weights, which were an average of two days on normal salt diet prior to starting high or low salt diets.

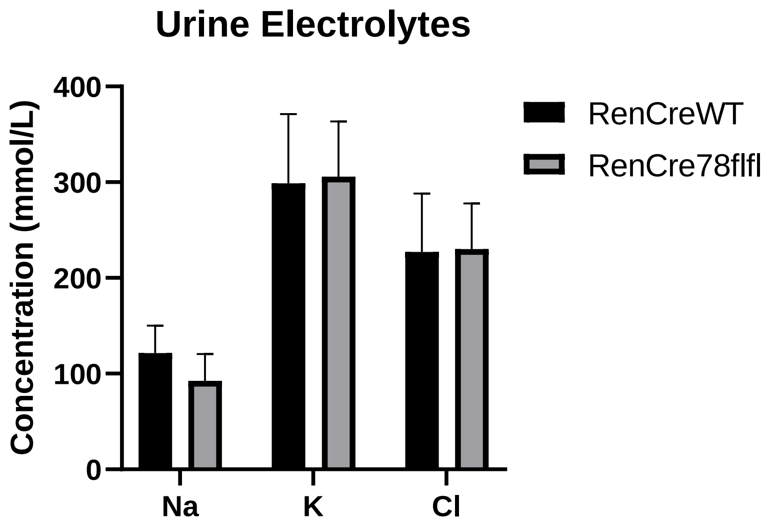

Supplemental Figure 3: Urine Electrolytes in ReninCre (WT) and ReninCreOlfr78$^{fl/fl}$ (KO) mice. Mice were kept in metabolic cages, and 24 hour urine samples were analyzed for sodium, potassium, and chloride levels. n=3 WT and KO. Each mouse was sampled for three days of urine electrolyte readings and averaged.

Parameter	ReninCre	ReninCreOlfr78flfl
**Na (mM)**	141.2±2.04	142±0.71
**Cl (mM)**	114.0±1.26	112.75±1.92
**TCO2**	17.4±1.74	18.5±2.50
**Glu (mg/dL)**	281.25±13.81	277.25±29.42
**BUN (mg/dL)**	33.6±2.58	30.0±3.24
**Crea (mg/dL)**	0.2	0.2
**Hct (%PCV)**	36.0±2.97	37.0±1.22
**Hb (g/dl)**	12.26±1.02	12.575±0.41

Supplemental Table 1: Plasma chemistry of ReninCre Mice: Blood chemistries of ReninCre(WT) and ReninCreOlfr78$^{fl/fl}$ (KO) mice. *n=5*, male (M) WT, n=4 M KO. TCO2, total carbon dioxide; BUN, blood urea nitrogen; PCV, packed cell volume.
